# Supplementary material for: Long-lasting vocal plasticity in adult marmoset monkeys
Source: Proc Biol Sci. 2019 Jun 26;286(1905):20190817. doi: 10.1098/rspb.2019.0817 (PMC6599999; doi:10.1098/rspb.2019.0817)
Supplement: Zhao_Supplementary Materials Combined_Rev2.pdf [file rspb20190817supp1.pdf]

Supplementary Materials

Long-lasting Vocal Plasticity in Adult Marmoset Monkeys

Lingyun Zhao, Bahar Boroumand Rad, Xiaoqin Wang

correspondence to: xiaoqin.wang@jhu.edu

Proceedings of the Royal Society B
DOI: 10.1098/rspb.2019.0817

*** This PDF file contains the following contents:**

- Supplementary Method
- Supplementary Discussion
- Supplementary Figures S1-S7 and Figure Legend
- Supplementary Tables S1-S4

Supplementary Method

Additional analysis regarding the spontaneous change in fundamental frequency

When further examining the spontaneous change in fundamental frequency, we found that there was a weak but significant negative correlation between the change in the fundamental frequency for adjacent calls and their call intervals in three of the four subjects (9606, 9001 and 62U, Supplementary Fig.S2). When two calls were produced with a very short interval, the second call was likely to have a higher fundamental frequency than that of the first one. When two calls were produced with a long interval, the second call was likely to have a lower fundamental frequency. This relationship may provide some information for the possible mechanisms underlying the spontaneous change in fundamental frequency.

Additional details for the behavioral paradigm and apparatus

The sound level of the perturbation signals was measured by a hand-hold sound level meter (Brüel & Kjær Type 2250, Nærum, Denmark) with a 1/2 inch prepolarized free field microphone (Brüel & Kjær Type 4189). The recording cage was placed 60 cm in front of Speaker 1. Mic 1 was placed 40 cm in front of the recording cage.

For subject 9001, there was a five-month interval between Perturbation 1 sessions and Baseline 2 sessions in which other studies were performed. Therefore, this subject is not included in the analysis of the long-lasting effect for the comparison between Baseline 2 sessions and Perturbation 1 sessions. For other subjects, all sessions were performed consecutively (Supplementary Fig.S3).

Additional details for data analysis

The raw acoustic recordings (vocalizations + perturbation signals) were de-noised using the referenced noise filtering method as described in previous work from our lab [1]. Since there were some variations in the actual delay of perturbation signals due to the computer system, we selected perturbed calls only if the actual perturbation signal delay was within a reasonable window (see "Perturbation signal delay window" in Supplementary Table S1).

We used an analysis window in the latter half of the phee phrase to calculate the fundamental frequency. The rationale is as follows. There may be transient changes to the fundamental frequency right after the start of a perturbation signal. We aimed to avoid the transient changes and measured the steady state fundamental frequency after the perturbation signal started. Therefore, we left a 0.2 second buffer period after the latest perturbation signal delay. In addition to that, there were also calls with shorter phrase length due to perturbation [2]. We selected calls that terminated at least 0.15 second after the end of the analysis window to avoid measuring the frequency variation towards the end of phrases. We calculated the call fundamental frequency by taking the average of the fundamental frequencies in 1ms bins within the analysis window (fundamental frequency in each bin was calculated using 10ms window for Fourier transform).

To characterize the relationship between the change of the fundamental frequency between adjacent calls and their time interval, we calculated the Pearson correlation between the change of fundamental frequency and the time interval in log-scale using data from each group of sessions (Supplementary Fig.S2).

To characterize frequency shift over time across sessions, we calculated the frequency shift with respect to Baseline 1 sessions (Fig.4B, Supplementary Figs.S3-S4). This is done as follows: the frequency shift of each session group is first calculated relative to the fundamental frequency profile of the session group directly preceding it (e.g. Perturbation 1 to Baseline 1, Baseline 2 to Perturbation 1 and Perturbation 2 to Baseline 2); then for a given group, the median frequency shift of each previous group was summed and added to the frequency shift of the current given group.

Supplementary Discussion

Control of vocal production by marmosets

A key question in the studies of non-human primates is to what extent they can control their vocal production. In humans, such an ability means being able to initiate a vocalization in response to behavioral situations, to modify spectrotemporal structures of vocalizations based on environmental cues or auditory feedback of one's own voice, and to learn to make changes in vocal production according to social contexts. Evidence for the control of the initiation of vocal production has been shown in tamarins and marmosets [1,3]. Evidence for the control of spectrotemporal structures of vocalizations, on the other hand, is scarce [2,4]. The observation of the bi-directional change in the fundamental frequency of phee calls (Fig.3, Supplementary Fig.S6) is one of the most intriguing findings in this study. Previous studies in non-human primates have described gross changes in vocal parameters such as amplitude and duration (e.g. Lombard effect) when perturbation signals were presented [5,6], which have largely been attributed to factors other than cognitive functions [4,7]. Beyond these gross changes, only one non-human primate study has shown evidence for spectral adjustment in vocalizations in response to noise perturbation [8]. In that study, researchers presented broadband or narrowband noise to cotton-top tamarins that overlapped with either all harmonics or the lower 3-4 harmonics of their combination long calls which had energy distributed over 6-8 harmonics. Several spectral parameters were modified in addition to an overall increase in vocal amplitude, which included an increase in peak frequency and a decrease in minimum frequency. However, that study did not dissociate spectral changes from the Lombard effect, nor did it explicitly test the direction of spectral changes (e.g., to include another noise type overlapping with higher harmonics of tamarin calls). It is therefore possible that the observed spectral change in vocalizations of cotton-top tamarins resulted from the change of vocal amplitude instead of a direct spectral modification. Studies in birds [9–11] and humans [12,13] also showed similar spectral changes accompanied by amplitude changes when tested with noise perturbation.

Effects in the second perturbation sessions

An interesting observation in our data is regarding the effect of the second perturbation sessions. The fundamental frequency of phee calls shifted in Perturbation 2 sessions (comparing to Baseline 2 sessions) in a similar manner as in Perturbation 1 sessions (comparing to Baseline 1 sessions), but the magnitude of the shift was relatively smaller (Fig. 4B). We performed a further analysis using the data from an example subject to illustrate how this smaller shift in fundamental frequency is related to the baseline shift after Perturbation 1 sessions (Supplementary Fig.S7). For the perturbation signal, the edge of its spectrum was chosen to be at the tail (±3 standard deviations) of the distribution of the fundamental frequency of phee calls in the previous baseline sessions. For Perturbation 1 sessions of this subject, the lower edge of the spectrum (cut-off frequency) of the high-frequency noise was positioned on the right side of the distribution of the fundamental frequency of phee calls measured in Baseline 1 sessions (8.842kHz, the left edge of the gray shaded area "P1" on Supplementary Fig.S7). If the baseline frequency had not shift, the distribution of the fundamental frequency in Baseline 2 sessions would have overlapped with that in Baseline 1 sessions (i.e., the cyan dashed curve overlaps with the blue curve in Supplementary Fig.S7). Therefore, we would have selected the upper spectral edge of the low-frequency noise based on the same distribution (7.197kHz, the gray vertical dashed line on Supplementary Fig.S7). However, because the fundamental frequency of Baseline 2 sessions shifted towards the lower frequency (Supplementary Fig.S7, the cyan dashed curve) and we assumed that the marmoset had already adopted this as the new baseline, we placed the upper spectral edge of the low-frequency noise based on this new fundamental frequency distribution (6.873kHz, the right edge of the gray shaded area "P2" on Supplementary Fig.S7). Therefore, relatively speaking, the spectrum of the low-frequency noise was placed farther away from the fundamental frequency distribution of the Baseline 1 sessions than the spectrum of the high-frequency noise. If the marmoset had already adopted the new baseline in Baseline 2 sessions, we would expect that the low-frequency noise had a similar effect size than the high-frequency noise because they were placed at the equal distance away from the spectrum of the baseline fundamental frequency distributions. A smaller effect size induced by the low-frequency noise suggests that the marmoset may have kept a memory of the original baseline (Baseline 1). In this case, using a perturbation signal with spectrum farther from this memorized baseline induced a smaller effect, as seen in all Perturbation 2 sessions (Fig.4). This observation provides further details on the long-term plasticity in the marmoset's vocal production system.

Neurophysiological implications

Findings from the present study also have implications on how the vocal control and plasticity are implemented in the marmoset brain. The traditional view on neural substrates of non-human primate vocal production is based on the notion that monkey vocalizations are largely emotional. In that case, neither voluntary nor feedback-dependent vocal control is needed for vocal production. Consistent with that notion, brain structures such as periaqueductal gray (a brainstem region) and anterior cingulate cortex are suggested to be responsible for producing vocalizations

in non-human primates (see reviews [14,15]). The cortical regions that are needed for planning and motor control such as lateral frontal cortex were thought to be dispensable [14,15]. More recent experiments using freely moving and vocalizing marmosets from our laboratory and others showed the involvement of premotor and prefrontal cortices in natural vocal production, providing new insights on the neural substrates of vocal control and planning [16,17].

The behavioral observations from the present study suggest the involvement of cognitive control in vocal production by marmosets. Therefore, several sensory and motor functions are expected to be engaged, including auditory monitoring of self-generated and environmental sounds, fine control of motor output parameters and the comparison between auditory information and planned motor production. These functions pose the necessity of frontal cortex and other sensorimotor cortices. For example, the auditory cortex is likely engaged in monitoring environmental sounds (e.g. perturbation signals and their spectra) and self-produced vocalizations (e.g. phee calls) [18–21]. Frontal regions, such as premotor and prefrontal cortex, may be engaged in the planning and control of vocal parameters (e.g. fundamental frequency) [16,17,22]. Interactions between these cortical areas are likely needed to calculate the direction and size of frequency shifts. Future studies will help delineate the cortical circuits underlying such voluntary control and plasticity in marmoset vocalizations.

References Cited in Supplementary Text

1. Roy S, Miller CT, Gottsch D, Wang X. 2011 Vocal control by the common marmoset in the presence of interfering noise. *J. Exp. Biol.* **214**, 3619–29. (doi:10.1242/jeb.056101)
2. Pomberger T, Risueno-Segovia C, Löschner J, Hage SR. 2018 Precise motor control enables rapid flexibility in vocal behavior of marmoset monkeys. *Curr. Biol.* **28**, 788-794.e3. (doi:10.1016/j.cub.2018.01.070)
3. Egnor SER, Wickelgren JG, Hauser MD. 2007 Tracking silence: adjusting vocal production to avoid acoustic interference. *J. Comp. Physiol. A* **193**, 477–483. (doi:10.1007/s00359-006-0205-7)
4. Ruch H, Zürcher Y, Burkart JM. 2017 The function and mechanism of vocal accommodation in humans and other primates. *Biol. Rev.* (doi:10.1111/brv.12382)
5. Brumm H. 2004 Acoustic communication in noise: regulation of call characteristics in a New World monkey. *J. Exp. Biol.* **207**, 443–448. (doi:10.1242/jeb.00768)
6. Eliades SJ, Wang X. 2012 Neural correlates of the lombard effect in primate auditory cortex. *J. Neurosci.* **32**, 10737–48. (doi:10.1523/JNEUROSCI.3448-11.2012)
7. Hotchkin C, Parks S. 2013 The Lombard effect and other noise-induced vocal modifications: insight from mammalian communication systems. *Biol. Rev. Camb. Philos. Soc.* **88**, 809–24. (doi:10.1111/brv.12026)
8. Hotchkin CF, Parks SE, Weiss DJ. 2015 Noise-induced frequency modifications of tamarin vocalizations: implications for noise compensation in nonhuman primates. *PLoS One* **10**, e0130211. (doi:10.1371/journal.pone.0130211)

9. Zollinger SA, Podos J, Nemeth E, Goller F, Brumm H. 2012 On the relationship between, and measurement of, amplitude and frequency in birdsong. *Anim. Behav.* **84**, e1–e9. (doi:10.1016/j.anbehav.2012.04.026)

10. Nemeth E, Pieretti N, Zollinger SA, Geberzahn N, Partecke J, Miranda AC, Brumm H. 2013 Bird song and anthropogenic noise: vocal constraints may explain why birds sing higher-frequency songs in cities. *Proc. R. Soc. London B Biol. Sci.* **280**.

11. Osmanski MS, Dooling RJ. 2009 The effect of altered auditory feedback on control of vocal production in budgerigars (Melopsittacus undulatus). *J. Acoust. Soc. Am.* **126**, 911–919. (doi:10.1121/1.3158928)

12. Lu Y, Cooke M. 2009 Speech production modifications produced in the presence of low-pass and high-pass filtered noise. *J. Acoust. Soc. Am.* **126**, 1495–1499. (doi:10.1121/1.3179668)

13. Garnier M, Henrich N. 2014 Speaking in noise: How does the Lombard effect improve acoustic contrasts between speech and ambient noise? *Comput. Speech Lang.* **28**, 580–597. (doi:10.1016/J.CSL.2013.07.005)

14. Jürgens U. 2002 Neural pathways underlying vocal control. *Neurosci. Biobehav. Rev.* **26**, 235–258. (doi:10.1016/S0149-7634(01)00068-9)

15. Jürgens U. 2009 The neural control of vocalization in mammals: a review. *J. Voice* **23**, 1–10. (doi:10.1016/j.jvoice.2007.07.005)

16. Roy S, Zhao L, Wang X. 2016 Distinct neural activities in premotor cortex during natural vocal behaviors in a New World primate, the common marmoset (Callithrix jacchus). *J. Neurosci.* **36**, 12168–12179. (doi:10.1523/JNEUROSCI.1646-16.2016)

17. Miller CT, Thomas AW, Nummela SU, de la Mothe LA. 2015 Responses of primate frontal cortex neurons during natural vocal communication. *J. Neurophysiol.* **114**, 1158–71. (doi:10.1152/jn.01003.2014)

18. Eliades SJ, Wang X. 2003 Sensory-motor interaction in the primate auditory cortex during self-initiated vocalizations. *J. Neurophysiol.* **89**, 2194–207. (doi:10.1152/jn.00627.2002)

19. Eliades SJ, Wang X. 2008 Neural substrates of vocalization feedback monitoring in primate auditory cortex. *Nature* **453**, 1102–6. (doi:10.1038/nature06910)

20. Eliades SJ, Tsunada J. 2018 Auditory cortical activity drives feedback-dependent vocal control in marmosets. *Nat. Commun.* **9**, 2540. (doi:10.1038/s41467-018-04961-8)

21. Eliades SJ, Wang X. 2013 Comparison of auditory-vocal interactions across multiple types of vocalizations in marmoset auditory cortex. *J. Neurophysiol.* **109**, 1638–57. (doi:10.1152/jn.00698.2012)

22. Nummela SU, Jovanovic V, de la Mothe L, Miller CT. 2017 Social context-dependent activity in marmoset frontal cortex populations during natural conversations. *J. Neurosci.* **37**, 7036–7047. (doi:10.1523/JNEUROSCI.0702-17.2017)

Supplementary Figures and Figure Legend

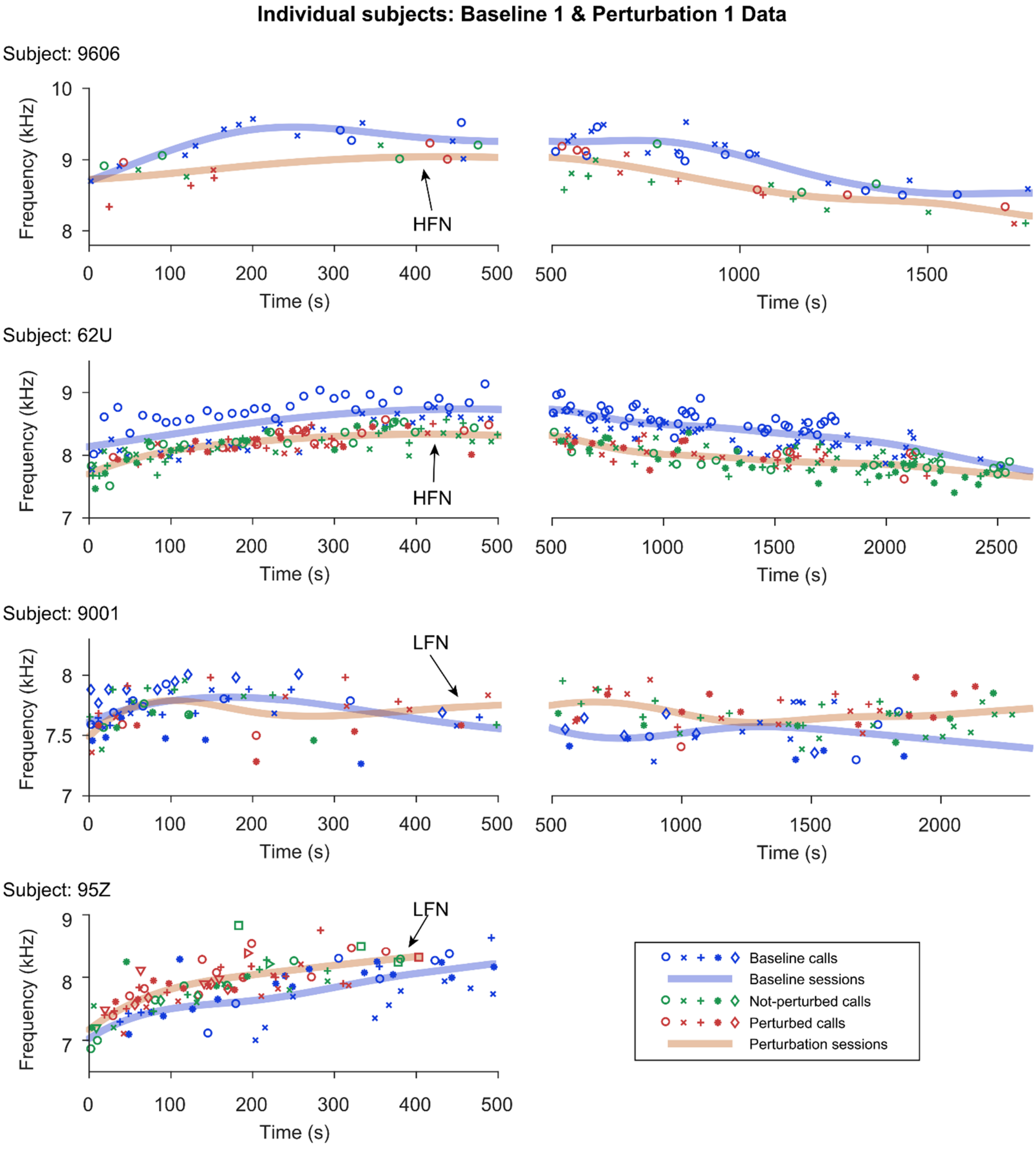

Fig. S1. Fundamental frequency data for individual experimental subjects (for Baseline 1 and Perturbation 1 sessions).

Similar format as Fig.3B. Fundamental frequencies with respect to call onset time for all four experimental subjects (Subject ID labeled at the top left corner of each row). Each marker indicates a call (same type of markers for calls in the same session). Blue: baseline; green: not-perturbed; red: perturbed. The fundamental frequency profile (fitted from individual data points) of baseline sessions is marked with a thick blue curve and that of perturbation sessions is marked with a thick orange curve. The x-axis is expanded for the first 500 seconds for a detailed view of the calls which are often more frequent at the beginning of each session. HFN: high-frequency noise; LFN: low-frequency noise.

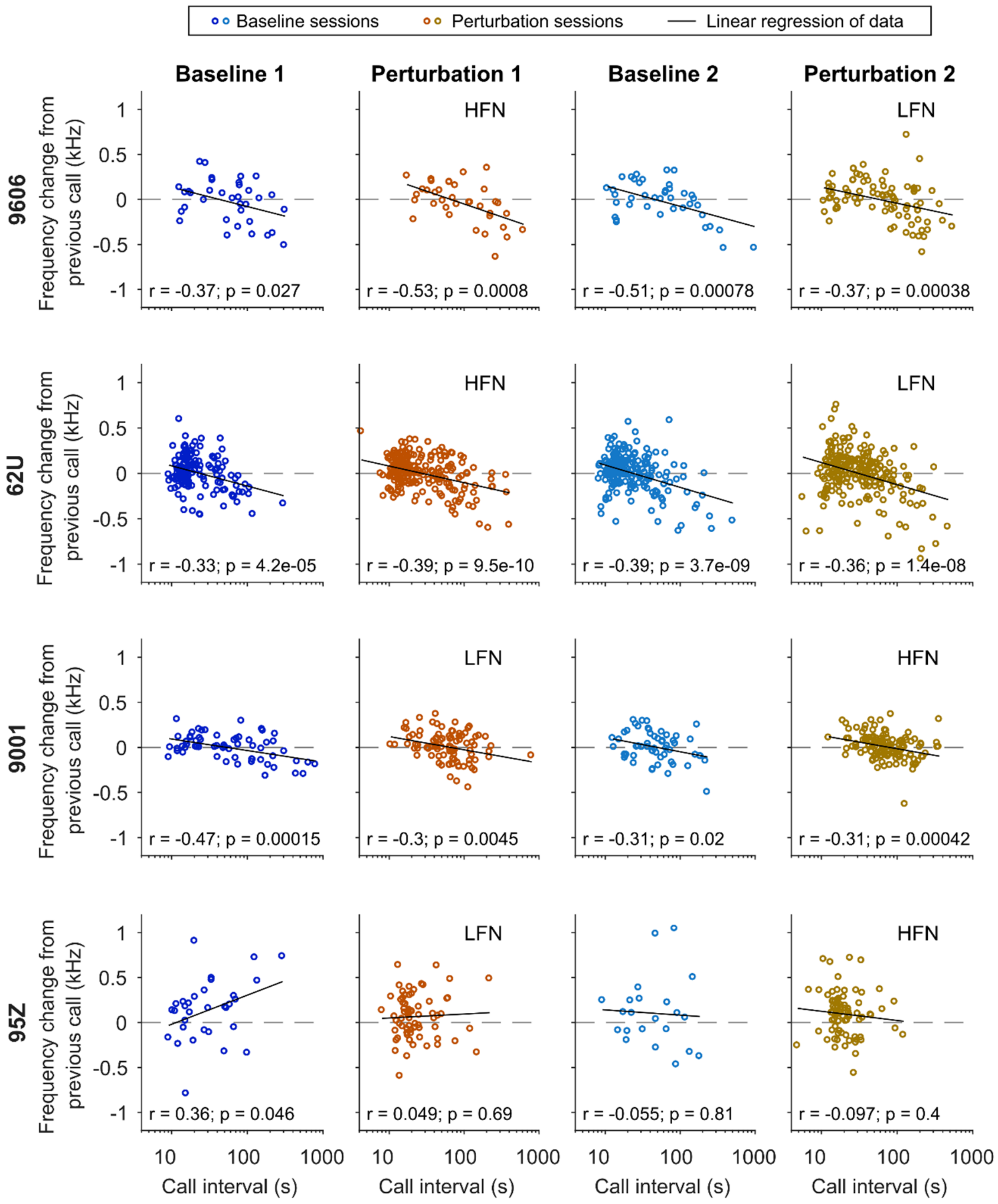

Fig. S2 Relationship of the frequency change between adjacent calls to the time interval between them.

Change in fundamental frequency between adjacent phee calls with respect to the call interval (onset-to-onset, in log-scale) for each experimental subject (Subject ID labeled on the left) in each of the experimental session groups (labeled on top). Data for the perturbation sessions include both the not-perturbed calls and the perturbed calls. The Pearson correlation is calculated, with the correlation coefficient and the p-value marked within each panel. Black line: a linear regression estimate of the data in each panel.

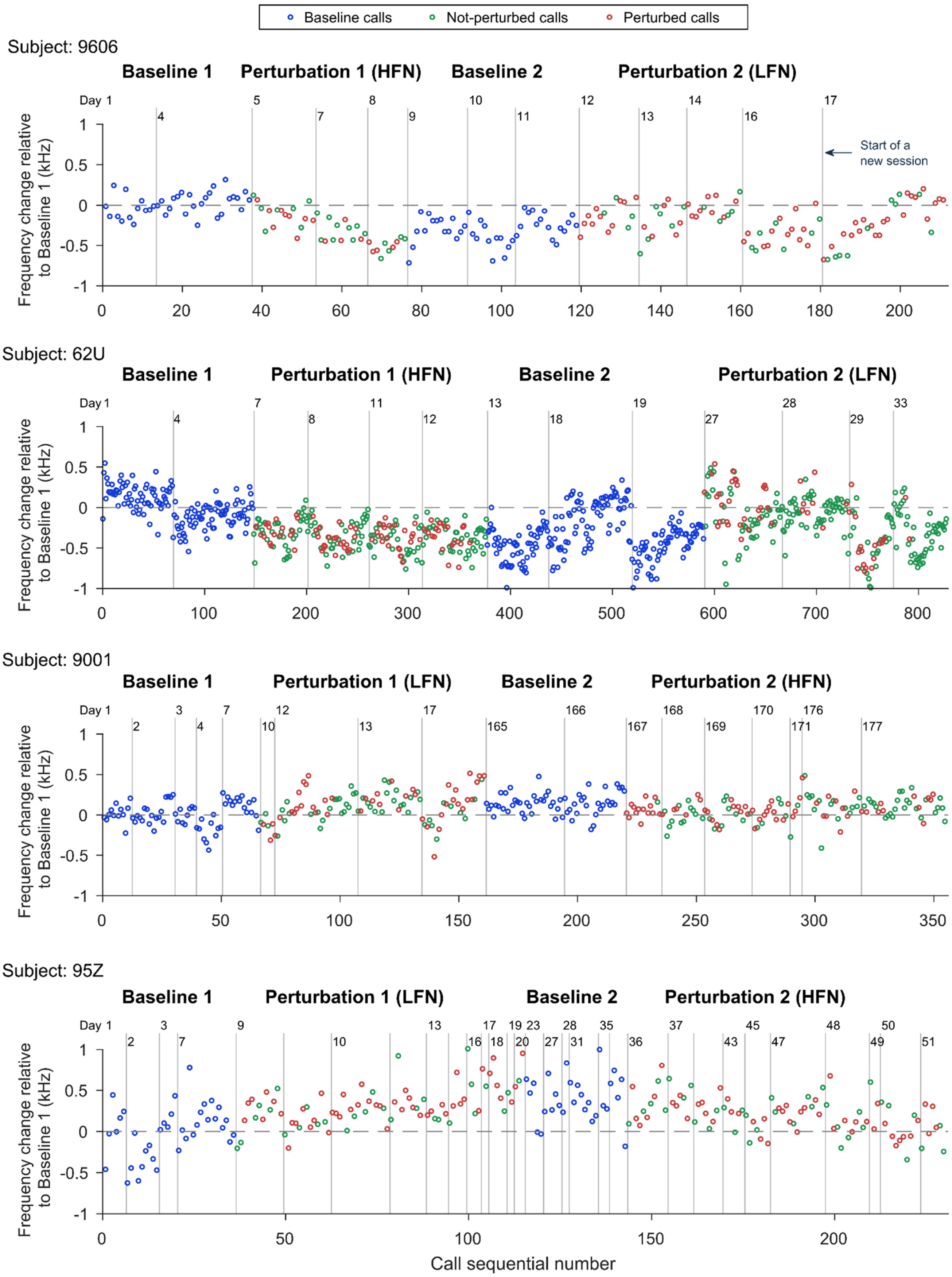
Baseline calls
Not-perturbed calls
Perturbed calls
Subject: 9606
Baseline 1
Perturbation 1 (HFN)
Baseline 2
Perturbation 2 (LFN)
Start of a new session
Subject: 62U
Baseline 1
Perturbation 1 (HFN)
Baseline 2
Perturbation 2 (LFN)
Subject: 9001
Baseline 1
Perturbation 1 (LFN)
Baseline 2
Perturbation 2 (HFN)
Subject: 95Z
Baseline 1
Perturbation 1 (LFN)
Baseline 2
Perturbation 2 (HFN)
Frequency change relative to Baseline 1 (kHz)
Call sequential number

Fig. S3 Relative frequency change of calls in temporal order across sessions.

Each data point represents the relative frequency change of a call in baseline sessions or in perturbation sessions, with respect to Baseline 1 sessions (see Methods). Blue: baseline calls; green: not-perturbed calls; red: perturbed calls. The vertical lines separate consecutive sessions. Data from each subject is plotted in a separate row (Subject ID labeled at the top left corner of each row). The experiment day is labeled on top of each panel, with the first session in Baseline 1 referred to as day 1. Test dates for all subsequent sessions are relative to day 1. A few data points outside of the [-1, 1] kHz range are plotted on the border of the y-axis.

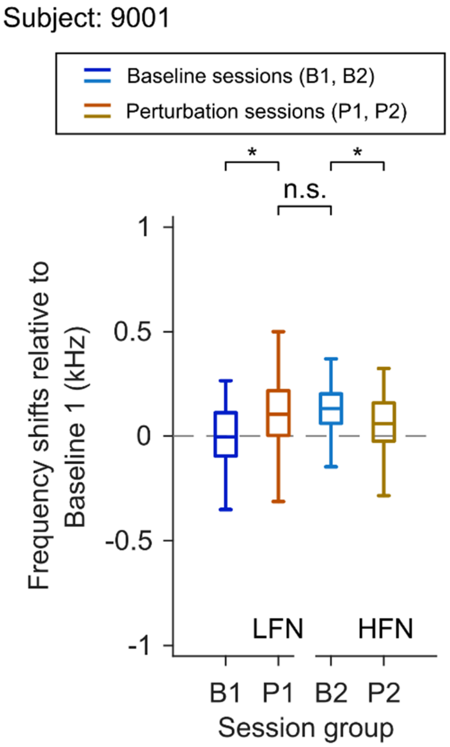

Fig. S4 Summary of frequency shifts in each session group for subject 9001.

Same format as Fig.4B. Fundamental frequency shifts (Tukey boxplot) over time in experimental session order, with respect to Baseline 1 sessions. Medians: horizontal lines inside the boxes. First and third quartiles: lower and upper borders of the boxes. Inner fences: whiskers outside of the boxes. Outliers are not plotted. Blue and cyan: baseline sessions. Orange and brown: perturbation sessions (including both the not-perturbed and the perturbed calls). B1: Baseline 1; P1: Perturbation 1; B2: Baseline 2; P2: Perturbation 2. Asterisks indicate significant differences. n.s. not significant. Kruskal-Wallis test results: $\chi^2 = 26.1$, $p = 9.2\times10^{-6}$. See Supplementary Table S3 for p-values in *post-hoc* analysis with Bonferroni corrections.

Individual subjects: Baseline 2 & Perturbation 2 Data

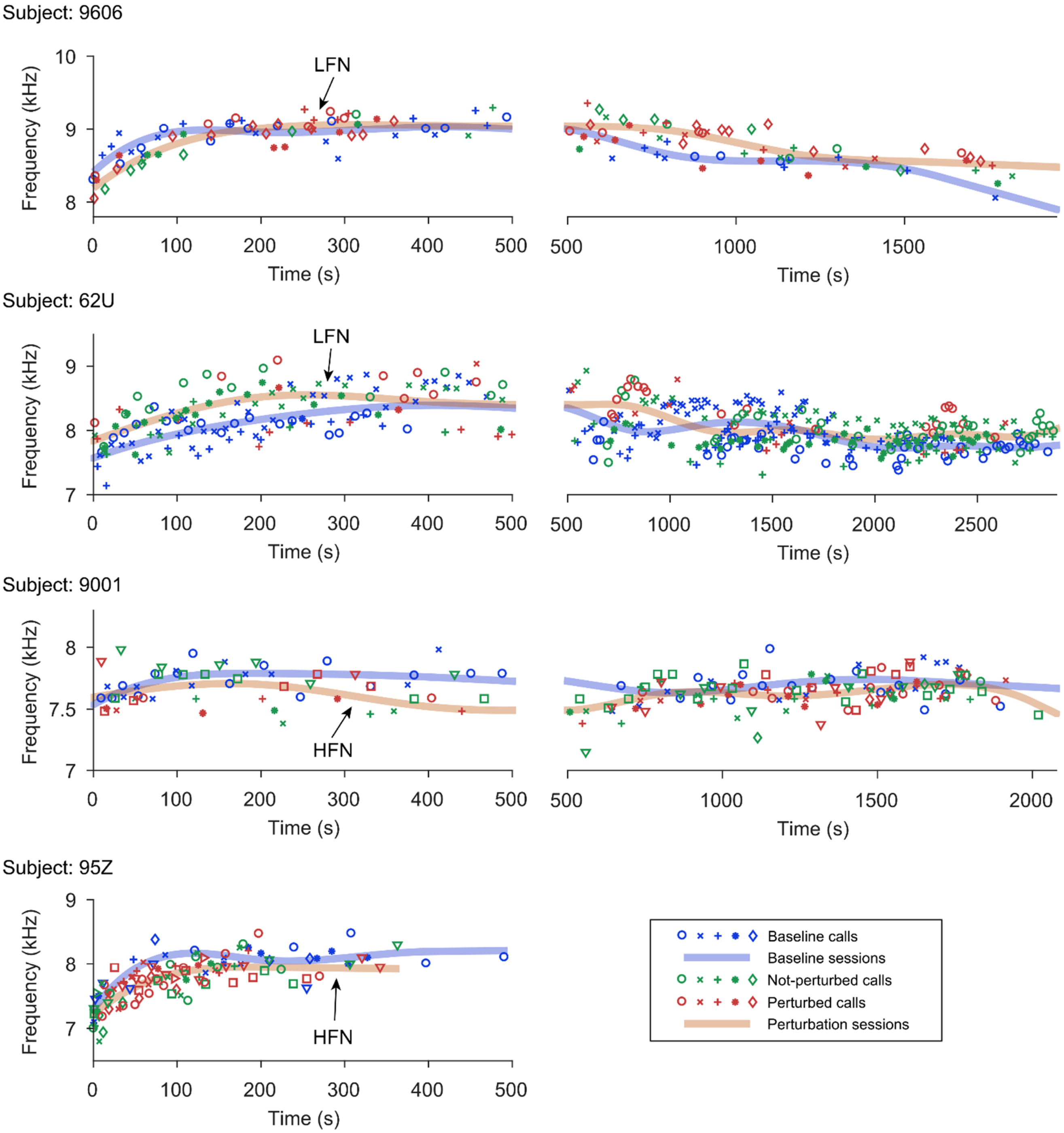

Fig. S5. Fundamental frequency data for individual experimental subjects (for Baseline 2 and Perturbation 2 sessions).

Similar format as Fig.3B. Fundamental frequencies with respect to call onset time for all four experimental subjects (Subject ID labeled at the top left corner of each row). Each marker indicates a call (same type of markers for calls in the same session). Blue: baseline; green: not-perturbed; red: perturbed. The fundamental frequency profile (fitted from individual data points) of baseline sessions is marked with a thick blue curve and that of perturbation sessions is marked with a thick orange curve. The x-axis is expanded for the first 500 seconds for a detailed view of the calls which are often more frequent at the beginning of each session. HFN: high-frequency noise; LFN: low-frequency noise.

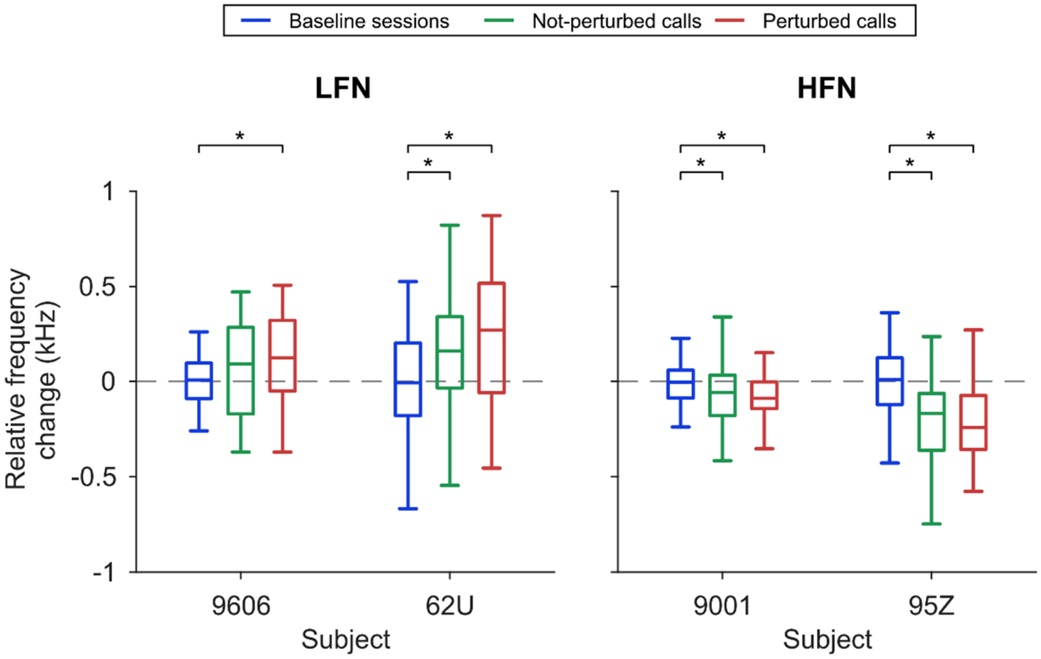

Fig. S6. The fundamental frequency of phee calls shifts away from perturbation signal spectrum in Perturbation 2 sessions.

Same format as Fig.3C. Statistical summary of the relative frequency change (Tukey boxplot) of calls in baseline (blue), not-perturbed (green) and perturbed (red) conditions for Baseline 2 and Perturbation 2 sessions (Left: LFN; Right: HFN). Medians: horizontal lines inside the boxes. First and third quartiles: lower and upper borders of the boxes. Inner fences: whiskers outside of the boxes. Outliers are not plotted. Any comparison showing a significant difference between conditions is indicated with asterisks. Kruskal-Wallis test results: Subject 9001: $\chi^2 = 13.3$, $p = 0.0013$, Subject 95Z: $\chi^2 = 17.0$, $p = 2.0\times10^{-4}$, Subject 9606: $\chi^2 = 8.8$, $p = 0.012$, Subject 62U: $\chi^2 = 36.2$, $p = 1.38\times10^{-8}$. See Supplementary Table S4 for the p-values in *post-hoc* analysis with Bonferroni corrections.

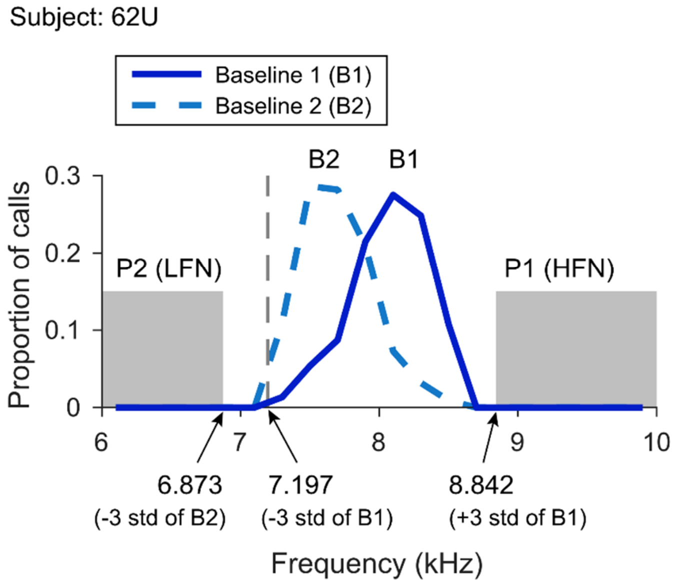

Fig. S7. Distribution of the fundamental frequency of phee calls in the baseline sessions and the position of the edges of the perturbation signal spectra (cut-off frequencies) for an example subject (62U).

Blue curve: Baseline 1 sessions ("B1"). Cyan dashed curve: Baseline 2 sessions ("B2"). The fundamental frequency is measured at 0.6s (half of the median phrase duration) after the call onset for the first phrase of phee calls (see Methods). Gray shaded areas illustrate the spectra of the perturbation signals in Perturbation 1 sessions ("P1", HFN) and Perturbation 2 sessions ("P2", LFN) to indicate the position of the spectral edge (cut-off frequency, the left side of the gray shaded area "P1" or the right side of the gray shaded area "P2"). The spectra of the perturbation signals are only partially shown (limited by the x-axis range). The gray vertical dashed line indicates the frequency three standard deviations below the mean of the fundamental frequency data from Baseline 1 sessions.

Supplementary Tables

Table S1. A list of parameters used in the experiment and analysis.

Subject		**9606**		**62U**		**9001**		**95Z**	
Perturbation signal type		HFN	LFN	HFN	LFN	HFN	LFN	HFN	LFN
Cut-off frequency (Hz)		10000	7500	8842	6873	8800	6000	8463	6623
Perturbation signal delay (s)		0.8		0.6		0.8		0.45	
Perturbation signal delay window (s)		[0.7, 0.9]		[0.5, 0.7]		[0.7, 0.9]		[0.35, 0.55]	
Analysis window (s)		[1.1, 1.3]		[0.9, 1.1]		[1.1, 1.3]		[0.75, 0.85]	
Total	$N_{Baseline}$	84	68	149	220	60	66	82	75
	$N_{Not\text{-}perturbed}$	44	69	148	195	69	50	41	64
	$N_{Perturbed}$	51	100	122	98	74	49	64	97
Analyzed	$N_{Baseline}$	37	43	148	213	59	66	29	36
	$N_{Not\text{-}perturbed}$	21	32	147	183	69	50	37	30
	$N_{Perturbed}$	18	60	82	55	66	45	50	49

Table S1 Legend

HFN: high-frequency noise; LFN: low-frequency noise. The cut-off frequency is the cut-off frequency of the high-pass or low-pass filters used to generate each type of perturbation signals. Perturbation signal delay is the program set parameter of the start time of perturbation signals relative to the onset of the first phrase of phee calls. Perturbation signal delay window is the allowed range of actual delay used in the analysis to include calls with reasonable perturbation signals. The analysis window is the time window for the first phrase of phee calls used to calculate the fundamental frequency. The "N" within the "Total" section is the number of calls in each condition recorded from the subject for each type of perturbation signals. The "N" within the "Analyzed" section is the number of calls selected and used in the analysis (according to perturbation signal delay window and other parameters, see Methods).

Table S2 P-values for the multiple comparison tests shown in Fig.3C

	Subject ID			
	**9606**	**62U**	**9001**	**95Z**
Perturbed vs. Baseline	1.3×10^{-6}	1.9×10^{-25}	9.2×10^{-4}	2.6×10^{-7}
Not-perturbed vs. Baseline	1.6×10^{-5}	7.6×10^{-34}	0.032	0.0010
Not-perturbed vs. Perturbed	1	1	0.86	0.64

Table S3 P-values for the multiple comparison tests shown in Fig.4B and Supplementary Fig.S4

	Subject ID			
	**9606**	**62U**	**9001**	**95Z**
Perturbation 1 vs. Baseline 1	5.0×10^{-9}	8.7×10^{-35}	8.3×10^{-4}	1.0×10^{-7}
Perturbation 2 vs. Baseline 2	0.021	6.6×10^{-8}	0.0074	3.9×10^{-4}
Baseline 2 vs. Perturbation 1	1	0.85	1	1

Table. S4 P-values for the multiple comparison tests shown in Supplementary Fig.S6

	Subject ID			
	**9606**	**62U**	**9001**	**95Z**
Perturbed vs. Baseline	0.0091	7.8×10^{-6}	0.0023	2.9×10^{-4}
Not-perturbed vs. Baseline	0.31	1.5×10^{-6}	0.0088	0.0018
Not-perturbed vs. Perturbed	0.99	0.55	1	1
